# Supplementary material for: Do Ophthalmic Solutions of Amphotericin B Solubilised in 2-Hydroxypropyl-γ-Cyclodextrins Possess an Extended Physicochemical Stability?
Source: Pharmaceutics. 2020 Aug 19;12(9):786. doi: 10.3390/pharmaceutics12090786 (PMC7559369; doi:10.3390/pharmaceutics12090786)
Supplement: Supplementary file 1 [file pharmaceutics-12-00786-s001.zip › Supplementary materials/Supplementary materials S1.docx]

Supplementary Materials: Do Ophthalmic Solutions of Amphotericin B Solubilised in 2-Hydroxypropyl-γ-Cyclodextrins Possess an Extended Physicochemical Stability?

Philip Chennell *, Mouloud Yessaad, Florence Abd El Kader, Mireille Jouannet, Mathieu Wasiak, Yassine Bouattour and Valérie Sautou

1. Validation of amphotericin B quantification method

The accuracy profile of the method used for amphotericin B quantification is presented Figure S1.

**Figure S1.** Accuracy profile of Amphotericin B validation. RTB: relative trueness bias. CI: confidence interval*.*

Representative chromatograms of amphotericin B solutions after forced degradation are presented Figure S2 (at 408 nm wavelength detection) and Figure *3* (diode array detection).


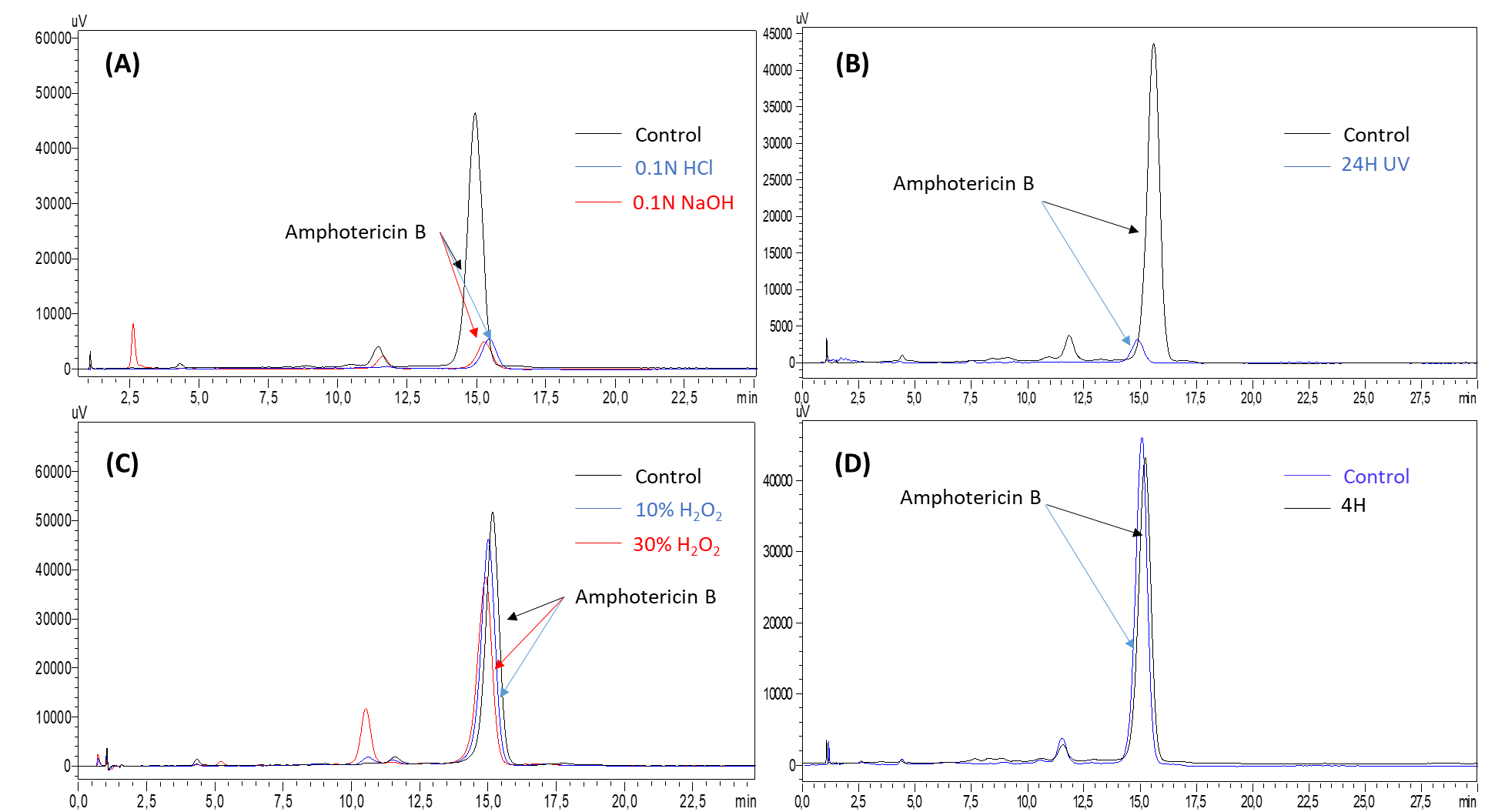


**Figure S2.** Chromatograms (at 408 nm wavelength detection) of amphotericin B solutions after forced degradation: (**A**) acid and alkaline exposure, (**B**) 24 h (24H) of ultraviolet-visible radiations exposure, (**C**) oxidative exposure and (**D**) heat exposure. 4H: after 4 h of heat exposure.


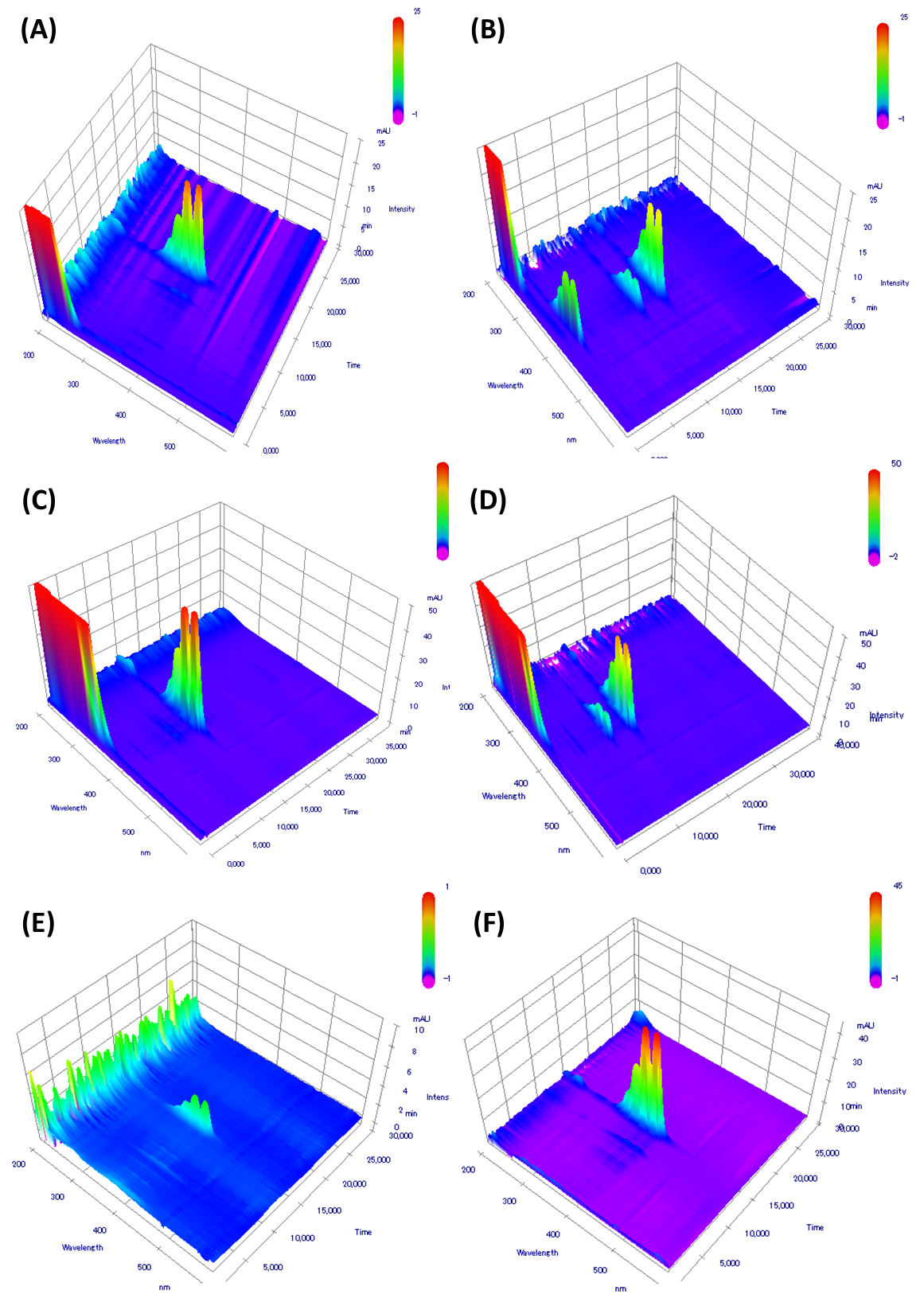


**Figure S3.**  Contour maps (diode array detection) of amphotericin B solutions after forced degradation: (**A**) 0.1N HCl, 1 h contact; (**B**) ) 0.1N NaOH, 1 h contact; (**C**) 10% H_2_0_2_, 2 h contact; (**D**) 30% H_2_0_2_, 2 h contact; (**E**) UV-visible radiations, 24 h contact; (**F**) heat exposure 60 °C, 4 h contact*.*

2. Identification of amphotericin B impurities

Chromatograms of the different impurities prepared following the European Pharmacopoeia Amphotericin B monography are presented Figure S4.


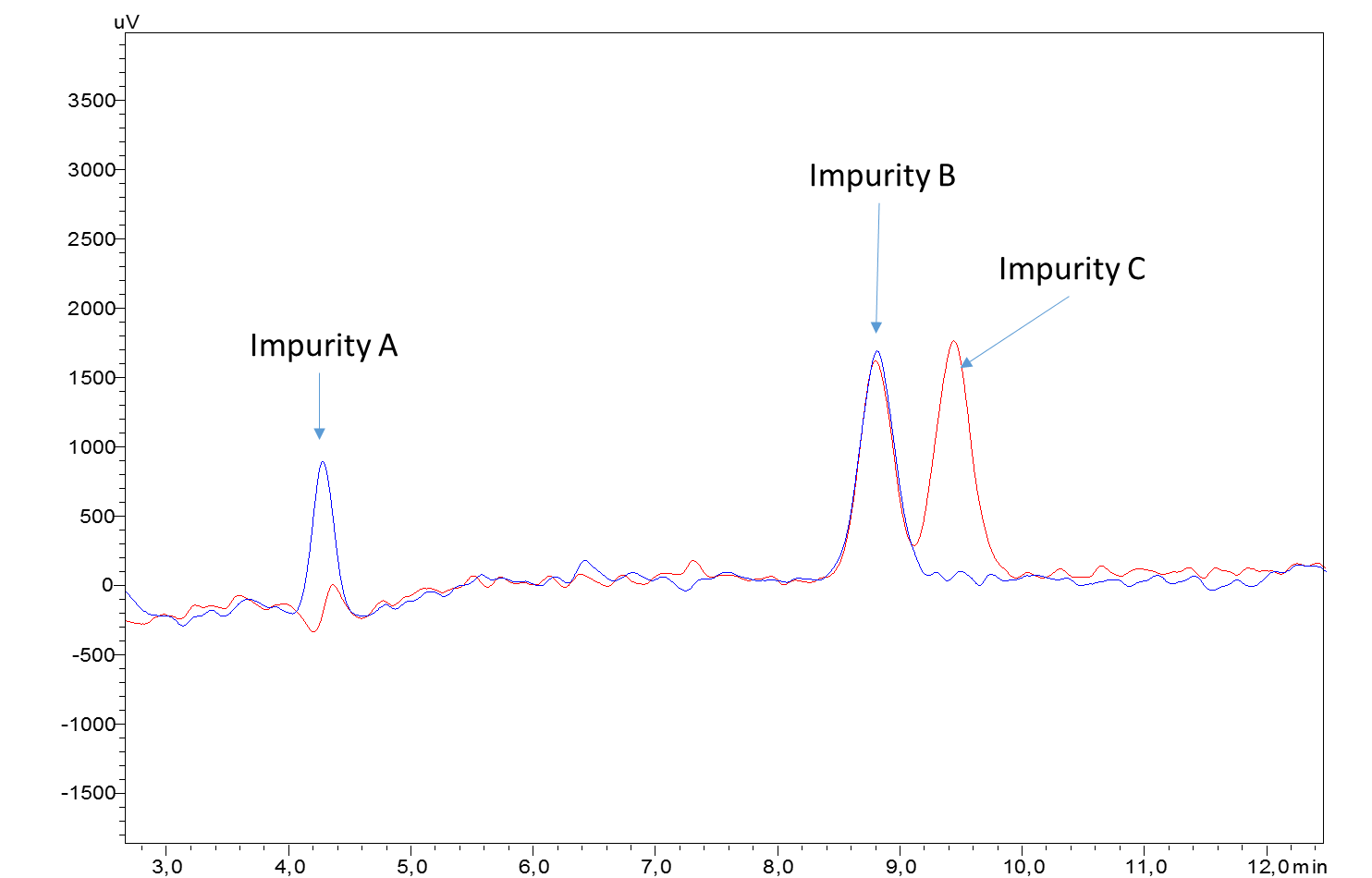


**Figure S4.** Chromatograms of amphotericin B impurities at 408 nm. Blue curve: amphotericin B for peak identification CRS containing impurities A and B; Orange curve: solution prepared following the procedure described in the amphotericin B monography allowing for the preparation of impurities B and C.

3. Visual aspect of ABDC and AB-HP-ɣ-CD formulations

An example of the visual aspect of the two formulations is presented Figure S5.


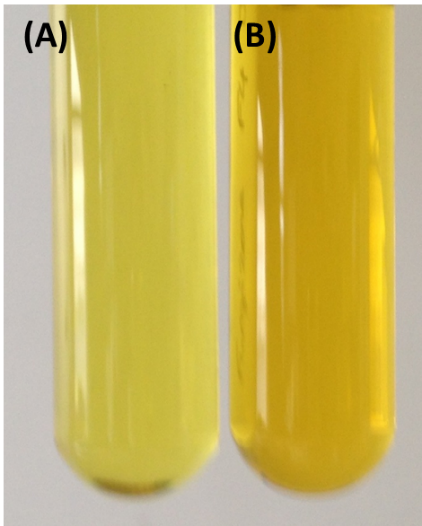


**Figure S5.** Visual aspect of the amphotericin B deoxycholate (**A**) and amphotericin B 2-hydroxypropyl-γ-cyclodextrin (**B**) formulations.
